# High-Temperature Properties of TC11-(TiB + TiC) Titanium Matrix Composites Fabricated by Hot Isostatic Pressing

**DOI:** 10.3390/ma19214519

**Published:** 2026-10-27

**Authors:** Yangyang Sun, Zhengfei Zhou, Zhongyang Mao, Yuyang Han

**Affiliations:** 1 School of Urban Science and Offshore Engineering, Nantong Institute of Technology, Nantong 226601, China; sunyangyang0526@163.com (Y.S.);; 2 Nanjing Shinkai Filtration Co., Ltd., Nanjing 211599, China; 3 College of Materials Science and Engineering, Nanjing Tech University, Nanjing 210009, China

**Keywords:** titanium matrix composites, hot isostatic pressure, high-temperature property

## Abstract

To meet the demand for lightweight, high-strength, and high-temperature-resistant structural materials in aerospace applications, TC11-(TiB + TiC) composites with varying B_4_C contents (0, 2 and 5 wt.%) were fabricated by hot isostatic pressing (HIP), and their microstructures and high-temperature mechanical properties were systematically investigated. Results reveal that in situ TiB whiskers and TiC particles were obtained in all the composites, and the matrix microstructure changed non-monotonically with the B_4_C content. A fine and uniform basketweave microstructure was obtained at 2 wt.% B_4_C, whereas at 5 wt.% the reinforcements formed a quasi-continuous network along the prior particle boundaries, and pores developed at the grain boundaries owing to B_4_C agglomeration and incomplete local reaction. At room temperature, the yield strength rose from 1020 MPa to 1586 MPa with increasing B_4_C, but the fracture strain dropped from about 37% to 11%. At 650 °C, however, the base alloy softened severely, its peak stress falling from 1998 MPa to 484 MPa. The composites, by contrast, were strengthened, with the peak stresses of TMC2 and TMC5 reaching 603 and 841 MPa, exceeding the base alloy by 24.6% and 73.8%, respectively. This reversal can be attributed to a change in the role of the reinforcements. At room temperature, the reinforcements act primarily as stress-concentration sites. At elevated temperatures, however, they function as a rigid load-bearing skeleton. Load transfer through this skeleton, together with the relaxation of interfacial stresses by matrix plasticity, accounts for the monotonic increase in high-temperature strength with increasing reinforcement content. The moderate CTE mismatch between the reinforcements and the matrix may provide a secondary contribution, as suggested by the increasing apparent microstrain from XRD and the stacking faults within TiB observed by HRTEM. In addition, the B27 crystal structure of TiB, its [010] growth habit, and the well-bonded symbiotic TiB/TiC interfaces may further impede dislocation motion at elevated temperature.

## 1. Introduction

Titanium alloys have been widely used in the aerospace, automotive, and energy industries owing to their excellent specific strength, good corrosion resistance, and outstanding biocompatibility [1,2]. However, titanium alloys, represented by Ti-6Al-4V (TC4), suffer from significant strength degradation and accelerated creep deformation at service temperatures exceeding 400–500 °C, which severely restricts their application in high-temperature components of advanced aero-engines [3,4]. Another important issue in the 400–500 °C range is oxidation: above ~480 °C, titanium alloys form a hard and brittle oxygen-enriched α-case beneath the oxide scale, which has been shown to degrade the yield strength and fatigue life of TC11 even after exposure at 500 °C [5,6]. For TiB/TiC-reinforced titanium matrix composites synthesized from B_4_C, well-dispersed (TiB + TiC) particulates have been reported to improve oxidation resistance and scale adherence, although the strong oxygen affinity of TiB whiskers also makes the oxidation behavior dependent on reinforcement morphology and distribution [7,8]. Therefore, extensive research efforts have been devoted to the development of novel high-temperature titanium alloys and titanium matrix composites (TMCs) with enhanced thermal stability and superior mechanical properties at high temperature.

Among various reinforcement strategies, in situ synthesized ceramic reinforcements, particularly TiB whiskers and TiC particles, have attracted considerable attention due to their excellent thermodynamic stability, favorable interfacial compatibility with the titanium matrix, and effective load-bearing capability at high temperatures [9,10]. The in situ reaction between B_4_C and Ti during powder metallurgy or casting processes enables the effective realization of TiB and TiC dual-phase reinforcement, giving rise to synergistic strengthening effects including load transfer and grain refinement [11,12]. Sun et al. [13] fabricated TMCs containing in situ TiB whiskers via plasma-activated sintering. The results demonstrate that the composite achieves a relative density of 98.33%, a Vickers hardness of 389.75 HV, and a maximum compressive yield strength of 1190 MPa, fully demonstrating the significant strengthening potential of in situ TiB whiskers. Recent studies have shown that, compared with the unreinforced base alloy, the TC4-(TiB + TiC) composite exhibits significantly improved high-temperature strength and creep resistance, which is attributed to the rigid skeleton structure formed by TiB whiskers and the pinning effect of TiC particles on dislocation motion at high temperatures [14,15]. 

Ti-6.5Al-3.5Mo-1.5Zr-0.3Si (TC11), a typical α + β dual-phase alloy with excellent high-temperature and room-temperature mechanical properties, has been widely used in critical components such as compressor disks and blades of aero-engines, with a maximum service temperature of approximately 500 °C [16,17]. Compared with TC4, TC11 contains a higher Si content along with alloying elements such as Mo and Zr, which facilitates the precipitation of ordered silicides, thereby enhancing the creep resistance and high-temperature strength of the alloy. However, when the service temperature exceeds 500 °C, the mechanical properties of TC11 still degrade significantly, making it difficult to meet the demands of next-generation aero-engines for higher operating temperatures. Therefore, it is imperative to further strengthen this alloy through the introduction of ceramic reinforcements.

Hot isostatic pressing (HIP) is a promising powder metallurgy technique that enables the fabrication of titanium matrix composites with homogeneous microstructures and minimal residual porosity [18,19]. Unlike conventional sintering, HIP applies uniform isostatic pressure from all directions at elevated temperatures, which effectively eliminates internal pores and promotes the formation of intimate interfacial bonding between the base alloy and the reinforcing phases [20,21]. Sun et al. [22] successfully fabricated TC4-TiC composites with a homogeneous microstructure with HIP and found that TiC particles exhibited superior strengthening effects compared to TiB whiskers at high temperature, demonstrating the potential of HIP in producing high-performance titanium matrix composites. Furthermore, Wang et al. [23] investigated TC4-B-Si titanium matrix composites, and revealed that a regularly arranged TiB whisker network was formed along the prior β-Ti grain boundaries, resulting in a 28.2% increase in microhardness compared to the monolithic TC4 alloy. These findings indicate that the combination of HIP processing and in situ reactions is highly effective in tailoring the microstructure and mechanical properties of titanium matrix composites.

Despite the progress made in TC4-(TiB + TiC) composites, systematic studies on the high-temperature compression behavior of TC11-(TiB + TiC) composites fabricated by HIP remain scarce. In particular, the effects of B_4_C content on the microstructural evolution and high-temperature mechanical response of TC11-based composites have not been fully elucidated. Moreover, our previous research [24] has primarily focused on the effect of Fe on the microstructural evolution of TC11-(TiB + TiC) +xFe composites, while neglecting their high-temperature mechanical properties. Therefore, in this work, TC11-(TiB + TiC) composites with varying B_4_C contents (0 wt.%, 2 wt.% and 5 wt.%) were fabricated by HIP using the in situ reaction between B_4_C and TC11 powder. The microstructural characteristics and compression properties at high temperature were systematically investigated. The findings are expected to provide valuable insights into the design and application of TC11-based composites for high-temperature structural components.

## 2. Materials and Methods

### 2.1. Materials

**Figure 1:**
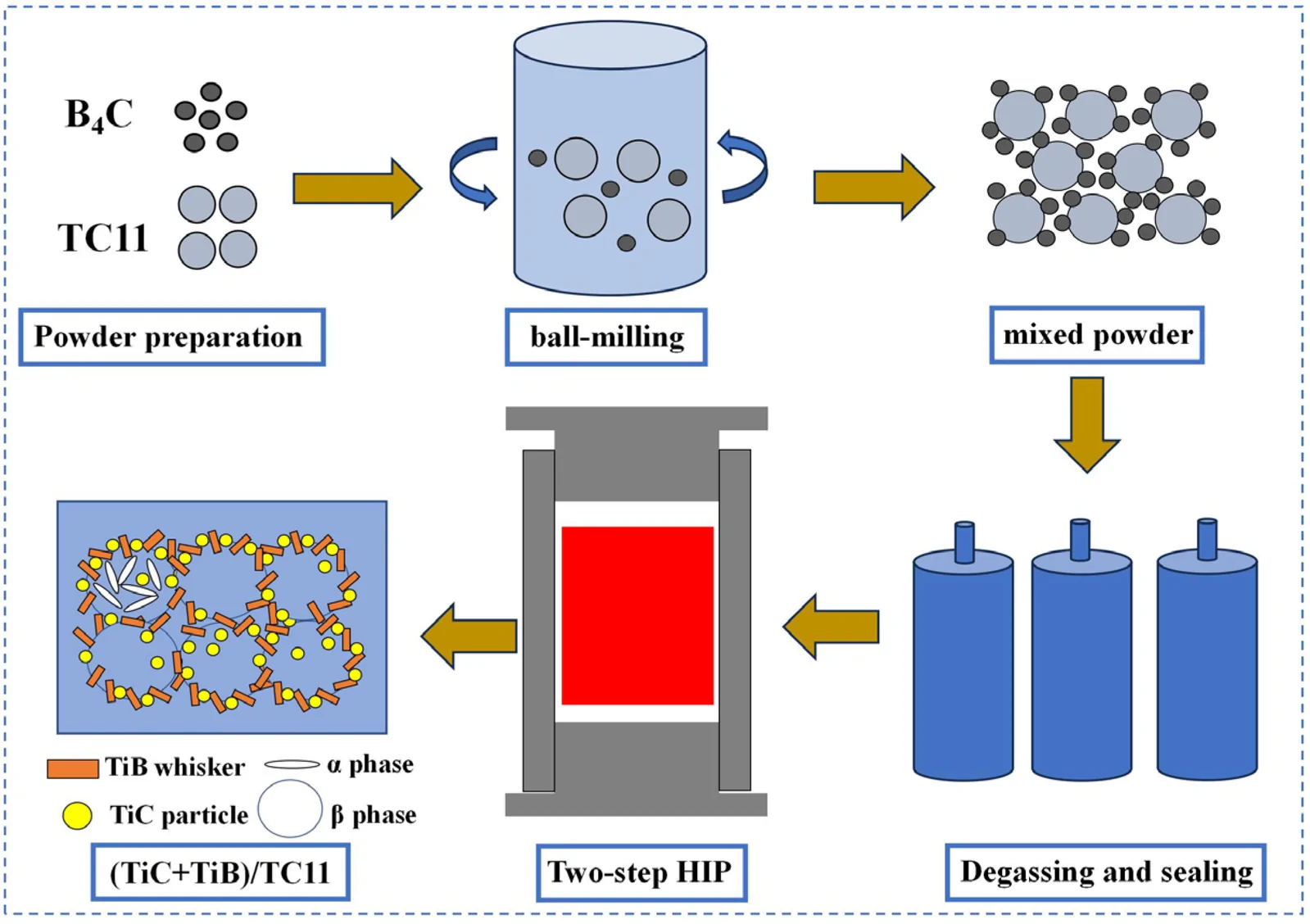
The Flow chart of TC11-(TiB + TiC) composites fabricated by HIP.

The base alloy used in this study was Ti-6.5Al-3.5Mo-1.5Zr-0.3Si (TC11) alloy powder with a normal particle size distribution ranging from 53 to 100 μm (99.9% purity), fabricated by the plasma rotating electrode process (PREP, Shangji Co., Nanjing, China). The ceramic reinforcement added was B_4_C powder with an average particle size of 50 nm, and different weight percentages (0 wt.%, 2 wt.%, and 5 wt.%). The TC11-(TiB + TiC) composites were fabricated in situ using HIP (QIH-9, Zhuye Huoju Metal Co., Zhuzhou, China) following the reactions 5Ti + B_4_C→4TiB + TiC and Ti + C→TiC. As shown in Figure 1, the TC11 and B_4_C mixed powders were first dried in a vacuum oven at 100 °C for 4 h and subsequently transferred to a planetary ball mill for low-energy ball milling with a rotation speed of 200 rpm for 6 h. The weight ratio of the agate ball to the powder mixtures was 4:1. The mixed ball-milled powders were sealed into a stainless-steel capsules with an inner diameter of 40 mm, a length of 150 mm, and a wall thickness of 2.5 mm. The encapsulated powders were then degassed at 600 °C for 6 h under a vacuum of 1 × 10^−3^ Pa.

The initial HIP sintering was performed at 1050 °C and 150 MPa for 4 h. The temperature of this cycle was deliberately kept below the Ti–Fe eutectic temperature (1085 °C) to prevent the reaction between the stainless steel capsule and the TC11 powder during densification. After cooling to room temperature, the stainless-steel capsule was mechanically removed. Subsequently, a two-step HIP sintering was conducted at 1150 °C and 150 MPa for 4 h to promote the further completion of the in situ reactions and enhance the densification. The higher temperature of the second cycle was intended to complete the in situ reaction between B_4_C and Ti, and to further densify and homogenize the composites. Ultimately, three different composite materials were prepared with different reinforcement ratios. The 0% B_4_C/TC11, 2 wt.% B_4_C/TC11 and 5 wt.% B_4_C/TC11 were labeled as TMC0, TMC2 and TMC5, respectively.

### 2.2. High-Temperature Compression Tests

The high-temperature compression tests of composite materials were performed at 650 °C using the electronic high-temperature tension-compression testing machine (HTZC-22014, Shangji Co., Nangjing, China) with a loading speed of 0.1 mm/min in accordance with Chinese national standard (GB/T 44030-2024, Metallic materials-High temperature compression test methods, China, 2024). Compression specimens were fabricated by wire cutting into standard cylindrical dimensions of Ø4 mm × 6 mm. Before testing, the ends were ground flat to ensure parallel contact surfaces. At least two tests were conducted for each composition, and the typical results were used to evaluate the high-temperature compression properties.

### 2.3. Microstructure Characterization

Phase identification was performed using X-ray diffraction (XRD, Rigaku SmartLab SE, AXT, Tokyo, Japan) with Cu Kα radiation (35 kV, 40 mA). The patterns were recorded in continuous scan mode from 30° to 80° (2θ) at a scanning speed of 10°/min. The original powders, mixed powders, and the microstructures of TMC0, TMC2 and TMC5 were observed using a scanning electron microscope (SEM, JEOL JSM-6490LV, Osaka, Japan). A transmission electron microscope (TEM, FEI Talos F200X, Thermo Fisher, Hillsboro, ON, USA) equipped with an energy dispersive spectrometer (EDS) was employed to characterize the base alloy, the distribution of reinforcement phases and elements, the microstructure morphology, and the interfacial bonding between the reinforcement phases and the base alloy in the titanium matrix composites. 

## 3. Results

### 3.1. Initial Microstructures

**Figure 2:**
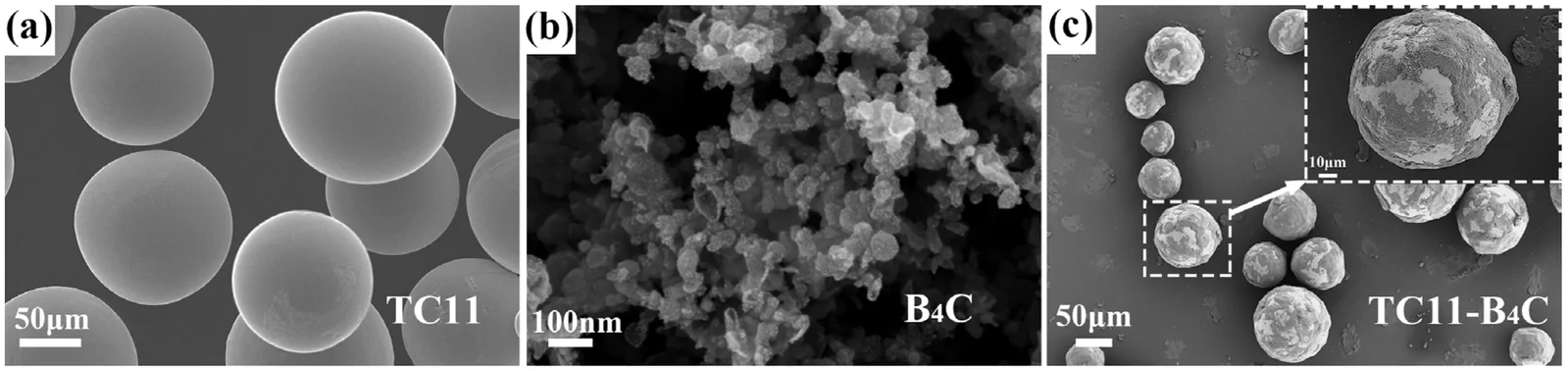
Morphology of powders: (**a**) TC11 powder; (**b**) B_4_C powder; (**c**) TC11-B_4_C mixed powder.

Figure 2 illustrates the morphology of the preform powders. Excellent sphericity and absence of visible defects is observed in both the pre-alloyed TC11 (Figure 2a) and mixed powders (Figure 2c), providing them with superior flowability. The B4C powder (Figure 2b) is uniformly attached to the surface of the pre-alloyed TC11 particles (Figure 2c). Additionally, as shown in Figure 2c, the magnified morphology of individual mixed powder particles reveals that a homogeneous combination of micron- and nano-scale particles is realized via low-energy ball milling. This allows the powder to vibrate and pack sufficiently into the stainless-steel capsules, facilitating the densification process.

**Figure 3:**
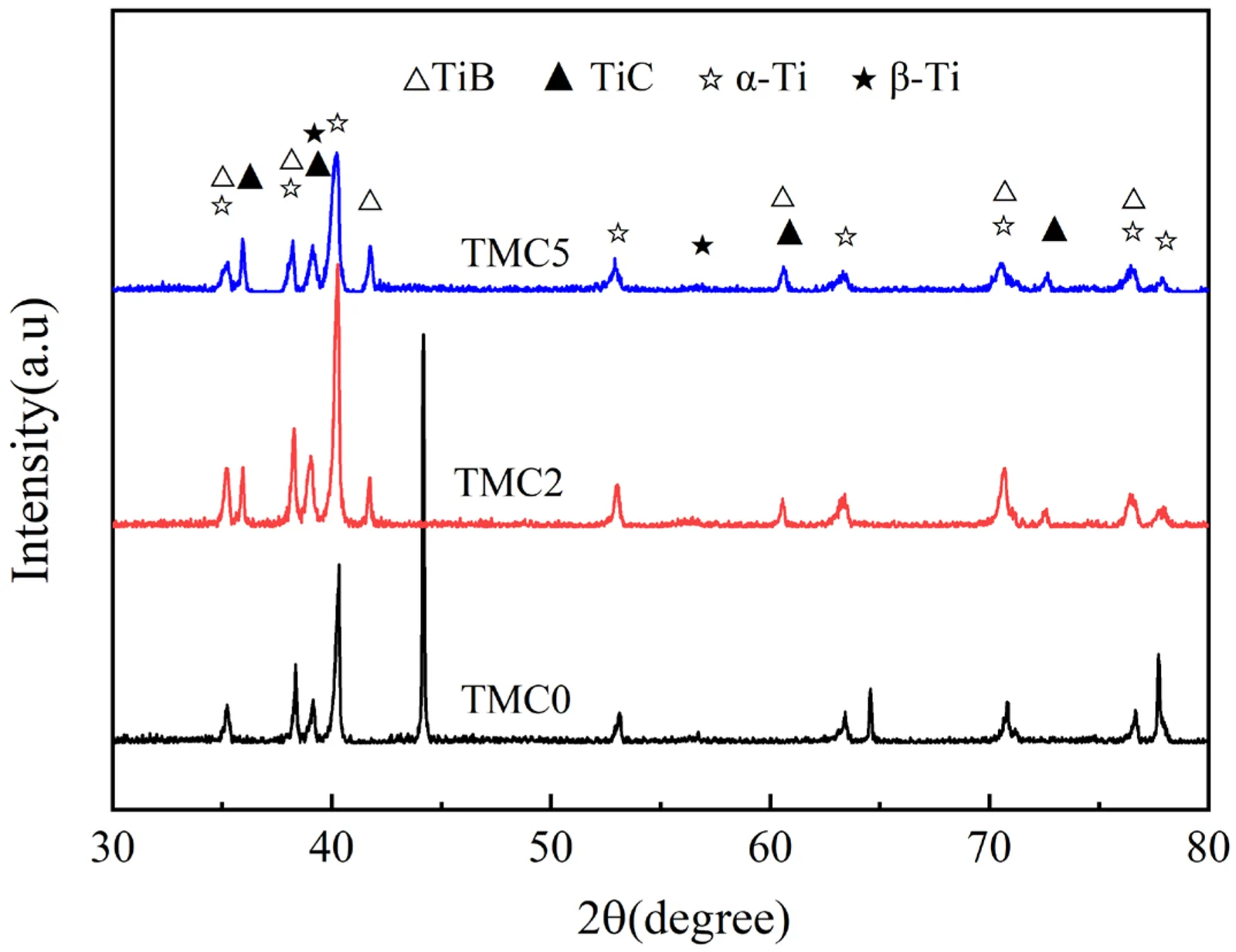
Analysis of XRD patterns for TMC0, TMC2, and TMC5.

Figure 3 presents the XRD patterns of the as-HIPed TMC0, TMC2, and TMC5 composites. Diffraction peaks of α-Ti, β-Ti, TiB, and TiC are clearly identified in all compositions, whereas no residual B_4_C reflections are detected, indicating that the in situ reaction (5Ti + B_4_C → 4TiB + TiC) proceeded to completion during HIP. This can be attributed to the high-temperature, high-pressure solid-state sintering environment of HIP and to the nanoscale size of the B_4_C powder, which favors the reaction kinetics. The intensity of the β-Ti reflections is low in all samples, indicating that only a small fraction of β phase precipitated. With increasing B_4_C content, the intensities of the TiB and TiC reflections progressively increase, consistent with the increasing volume fraction of the reinforcements. In addition, the α-Ti matrix reflections shift slightly toward lower 2θ values, ascribed to the partial solid solution of B and C atoms in the TC11 base alloy and the resulting small increase in the lattice parameters [25]. Concurrently, the α-Ti peaks exhibit monotonic broadening: the full width at half maximum (FWHM) of the (002) reflection increases from 0.091° to 0.160° and 0.290° for TMC0, TMC2, and TMC5, respectively, and the corresponding apparent microstrain [26,27] rises from 1.9 × 10^−3^ to 2.5 × 10^−3^ and 3.7 × 10^−3^. This increasing lattice microstrain is consistent with the thermal-expansion mismatch between the in situ formed TiB/TiC reinforcements and the TC11 matrix. No diffraction peaks of any impurity phases are observed, confirming that the composites consist only of the TC11 base alloy and the in situ-formed TiB and TiC reinforcements.

**Figure 4:**
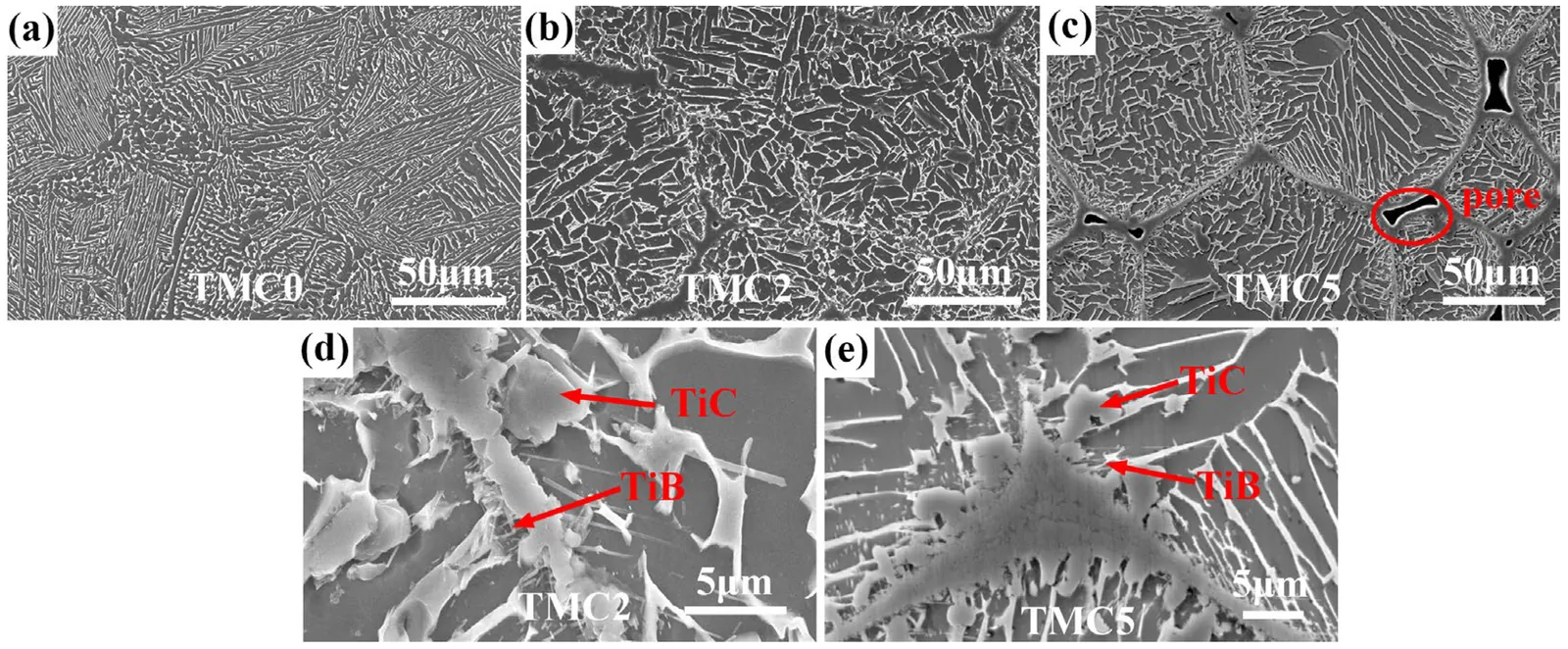
SEM micrographs of TC11-(TiB + TiC) composites: (**a**) TMC0, (**b**) TMC2, (**c**) TMC5; (**d**,**e**) higher-magnification images of TMC2 and TMC5 showing the in situ-formed TiB whiskers and TiC particles.

Figure 4 exhibits the microstructures of the as-HIPed TC11-(TiB + TiC) composites. In TMC0 (Figure 4a), the TC11 alloy consists of α laths organized into coarse, aligned colonies with an average size of approximately 70 μm. At 2 wt.% B_4_C (TMC2, Figure 4b), a fine, uniform basketweave matrix forms, with a median α-lath width of 2.5 μm. Fine needle-like TiB whiskers are distributed along the interfaces, accompanied by near-equiaxed or blocky TiC particles within the matrix grains or near grain boundaries, identified by their characteristic morphologies, the XRD results (Figure 3), and the elemental mappings (Section 3.3), which show discernible B enrichment at the whiskers and C enrichment at the blocky particles [24,28]. Both are well bonded to the base alloy without interfacial debonding or reaction layers. Higher-magnification images (Figure 4d,e) clearly resolve the whiskers and particles, which are too fine to be distinguished at the magnifications of Figure 4a–c. At 5 wt.% B_4_C (TMC5, Figure 4c), the reinforcements are densely stacked along the prior particle boundaries, forming a quasi-continuous network, and the prior β grains coarsen to 70~130 μm with grain-boundary pores developing within the reinforcement-rich regions (circled in Figure 4c).

Quantitative image analysis gives a porosity fraction of 1.1~2.7% for TMC5, versus below 0.3% for TMC0 and TMC2. The pores are few but large (median equivalent diameter ≈ 5.4 μm, maximum ≈ 22.3 μm for pores ≥ 1 μm^2^) and preferentially located at grain boundaries and triple junctions where the reinforcements are densely stacked. This distribution indicates that the pores originate from B_4_C agglomeration and incomplete local interfacial reaction rather than insufficient densification. The agglomerates cannot be fully consumed in the solid-state reaction and leave local volume deficits, while the surrounding rigid TiB/TiC network impedes the matrix plastic flow required for pore closure under HIP pressure. Accordingly, the uniformly distributed sub-micron porosity that HIP normally eliminates is absent in TMC0 and TMC2.

The B_4_C content exerts a non-monotonic influence on the matrix microstructure. At 2 wt.% B_4_C, grain refinement is observed. This refinement may arise from heterogeneous nucleation of β grains on in situ-formed TiB/TiC during cooling through the β-transus. It may also be assisted by Zener pinning of grain boundaries by the reinforcement network, and by solute drag from B and C atoms ahead of the growing grains. At 5 wt.% B_4_C, particle agglomeration during blending likely reduces the number of effective nucleation sites. The increasingly dense, quasi-continuous reinforcement network may then promote local grain coarsening and grain-boundary porosity. 

### 3.2. High-Temperature Property

**Figure 5:**
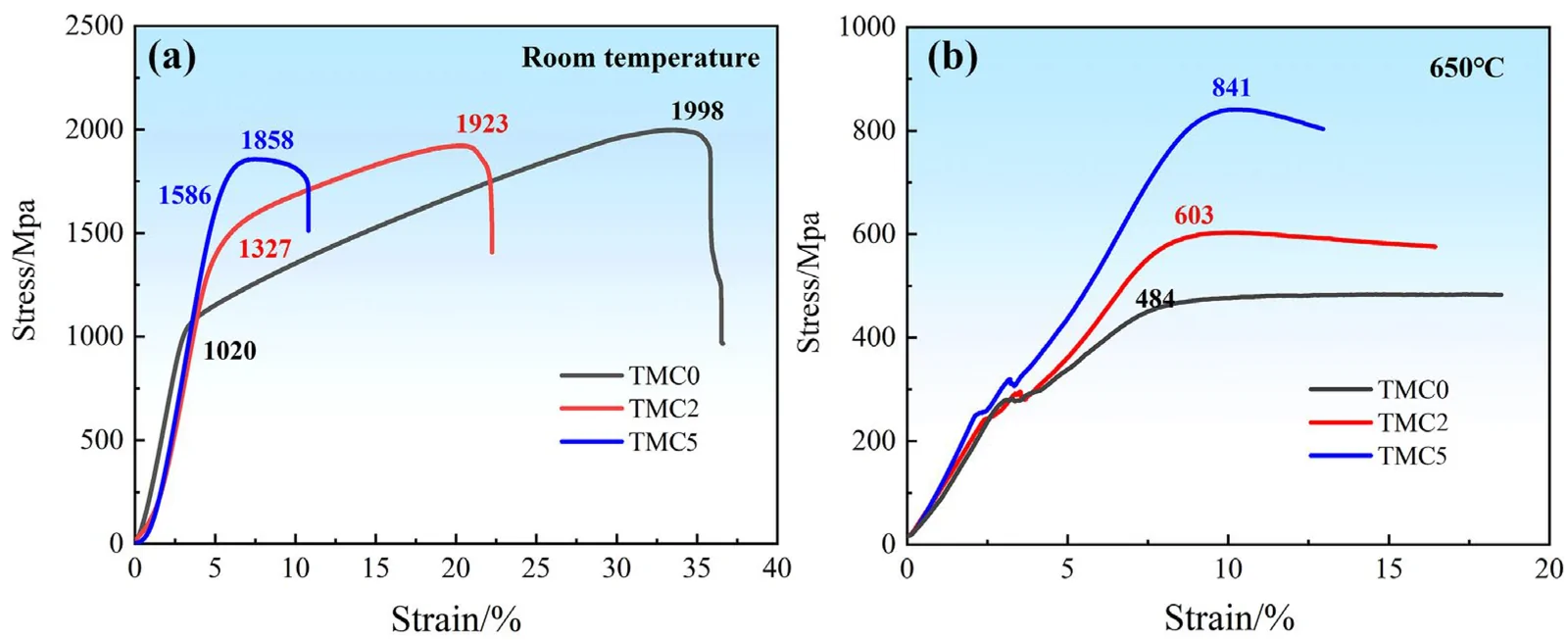
Compressive stress–-strain curves of TC11-(TiB + TiC) composites under (**a**) room temperature [24] and (**b**) high temperature of 650 °C.

Figure 5 displays the compressive properties of TC11-(TiB + TiC) composites at room temperature and high temperature of 650 °C. Room-temperature compression test results (Figure 5a) show that TC11 exhibits an excellent strength and ductility combination, with a compressive yield strength of approximately 1020 MPa and compression strain of about 37%. Upon the addition of B_4_C, the yield strength of the composites increases significantly. TMC2 exhibits a yield strength of approximately 1327 MPa, representing a 30.1% improvement over the base alloy, while TMC5 further increases to approximately 1586 MPa, corresponding to a 55.5% enhancement. These results confirm that the in situ formed TiB/TiC reinforcing phases exert a pronounced load transfer effect on the base alloy.

However, both the peak stress and ductility of the composites decrease progressively with increasing B_4_C content. The peak stress drops from 1998 MPa for the TC11 alloy to 1923 MPa for TMC2 and 1858 MPa for TMC5, while the compression strain decreases substantially from approximately 37% for the TC11 to about 22% for TMC2 and 11% for TMC5. This indicates that, although a high reinforcement content significantly enhances the yield strength, the presence of pores, TiB whisker clustering, and brittle network structures serve as preferential sites for crack initiation, leading to premature unstable fracture of the material and preventing the reinforcing phases from fully realizing their load-bearing potential.

For high-temperature compressive properties (Figure 5b), in the initial stage of plastic deformation, both the TC11-(TiB + TiC) composites and the TC11 base alloy exhibit a rapid increase in flow stress with increasing strain, demonstrating a pronounced work hardening behavior [29]. Since the composites are dominated by strain hardening during compression, the peak stress corresponds to the maximum compressive strength. The strengthening effect of the TC11-(TiB + TiC) composites exhibits a fundamental reversal compared to that at room temperature. At high temperature, the TC11 undergoes significant softening, with the peak stress decreasing sharply from 1998 MPa at room temperature to 484 MPa (a reduction of approximately 75.8%), accompanied by a substantial drop in yield strength, indicating that the base alloy has largely lost its primary load-bearing capacity at high temperature. In contrast, the composites exhibit a considerably smaller reduction in strength. The peak stresses of TMC2 and TMC5 are 603 MPa and 841 MPa, representing improvements of 24.6% and 73.8% over the base alloy, respectively.

Notably, the compressive strength of TMC5 at high temperature reaches 1.74 times that of the base alloy, whereas at room temperature it is only 93% of the base alloy value, exhibiting a pronounced reversal in the strength comparison. The underlying mechanism for this reversal is attributed to the excellent high-temperature elastic modulus and thermal stability of the TiB whiskers and TiC particles. When the base alloy softens and its load-bearing capacity diminishes significantly at high temperature, the reinforcing phases transform from stress concentration sites at room temperature into a rigid load-bearing skeleton, effectively transferring the applied load to the reinforcing phases through the load transfer mechanism, thereby significantly suppressing the overall plastic flow and strength degradation of the material.

### 3.3. Microstructural Evolution

**Figure 6:**
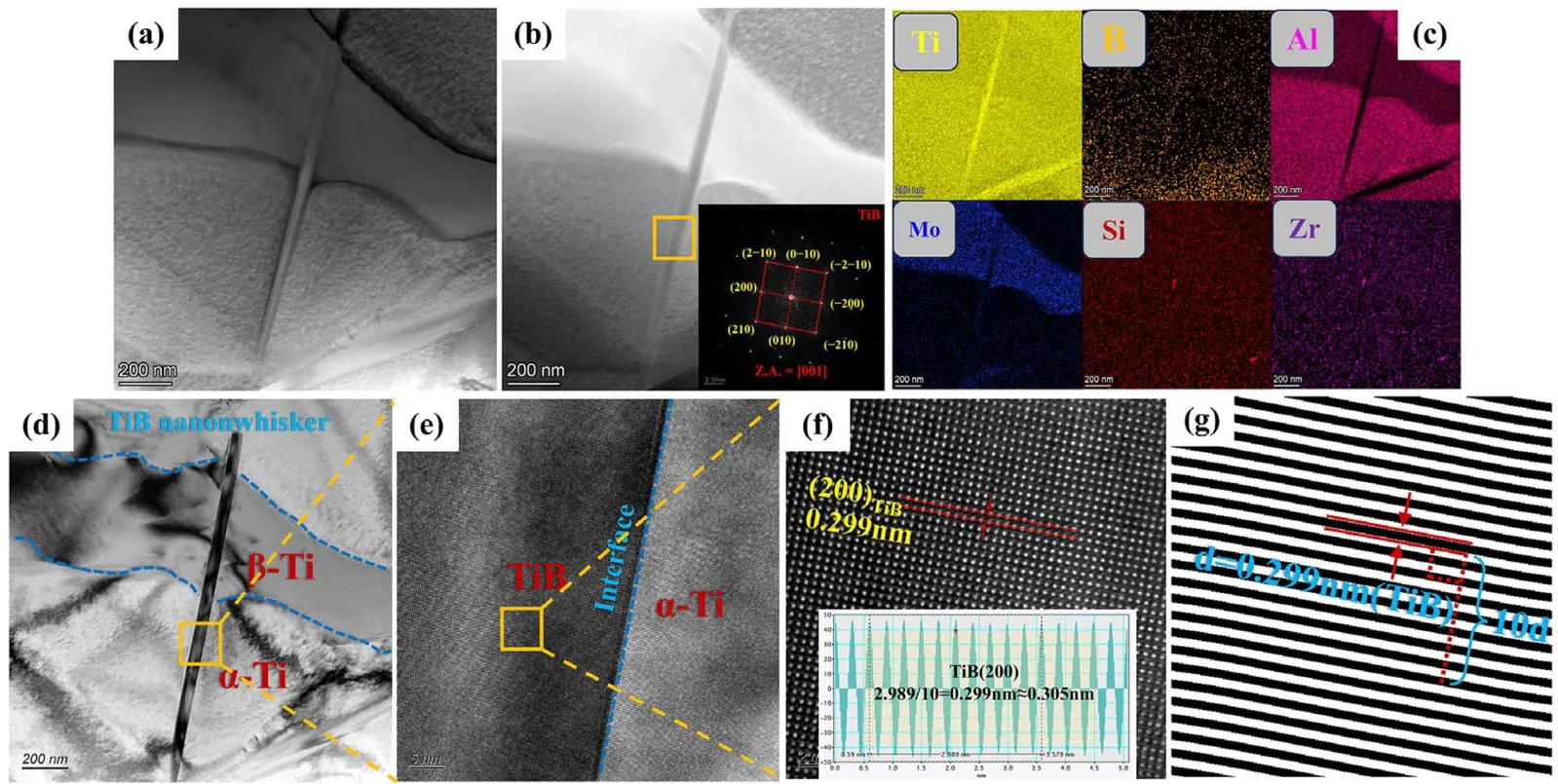
TEM characterization of in situ TiB whisker in TMC5: (**a**) micrograph of TiB whisker, (**b**) HAADF image of TiB whisker and SAED pattern, (**c**) elemental mapping images, (**d**) nanoscale TiB whisker, (**e**) HRTEM image of the interface between TiB and α-Ti in the selected region, (**f**) magnified HRTEM image of the selected region, (**g**) IFFT image obtained from the HRTEM image.

Figure 6 presents the TEM image of TiB whiskers precipitated within the α-Ti and β-Ti phases in the TMC5. It can be observed that the nanowire-like TiB whiskers are distributed throughout both the α-Ti and β-Ti phases (Figure 6a). Figure 6b shows the corresponding selected area electron diffraction (SAED) pattern indexed along the [001] zone axis, which further confirms the simple lattice crystal structure of TiB with an orthorhombic system. Figure 6c displays the elemental mapping of Ti, Al, Mo, Si, Zr, and B in the TC11. Since B is a light element with a low atomic number, its characteristic X-ray emission energy is relatively low (0.183 keV) and susceptible to background noise interference. Consequently, the elemental map of B exhibits weak contrast. Nevertheless, an enrichment tendency of B at the TiB whiskers can still be discerned. Combined with the elemental mapping of Ti, the precipitated phase can be further identified as TiB. The Mo-enriched regions correspond to the β-Ti phase, while the Al-enriched regions correspond to the α-Ti phase. Si and Zr, as matrix alloying elements, are uniformly dissolved in the composite matrix.

Figure 6d presents the TEM bright-field image of TiB whiskers penetrating through the β-Ti and α-Ti matrix, revealing a clean and well-defined interface between the reinforcing phase and the base alloy. This indicates that the composite fabricated by hot isostatic pressing sintering possesses a favorable interfacial bonding quality, which is beneficial for maintaining excellent mechanical properties. Figure 6e shows the high-resolution transmission electron microscopy (HRTEM) image of the region marked by the rectangle in Figure 6d, further displaying the clean and well-defined interface between TiB and the α-Ti matrix, indicating that the in situ synthesized TiB whiskers form a strong interfacial bond with the base alloy. Figure 6f presents the high-magnification HRTEM image of the corresponding region, and Figure 6g shows the corresponding inverse fast Fourier transform (IFFT) image. Well-defined lattice fringes can be observed in the IFFT image, with an interplanar spacing of approximately 0.299 nm, corresponding to the (200) TiB. 

In addition, it is worth noting that TiB possesses a B27-type orthorhombic crystal structure, in which the Ti atoms are arranged in a layered configuration and the B atoms are distributed along one-dimensional chains. This unique structure endows TiB with high hardness, high melting point, and excellent chemical stability, making it widely applicable in the fields of composites and high-temperature-resistant materials. This also accounts for the significant improvements in both high-temperature yield strength and high-temperature compressive strength observed in the compression tests at elevated temperature after the introduction of a substantial amount of B element. Furthermore, in the B27 structure, the growth rate of B atoms along the [010] direction is considerably higher than that along other crystallographic directions, resulting in the whisker-like morphology of TiB [30].

**Figure 7:**
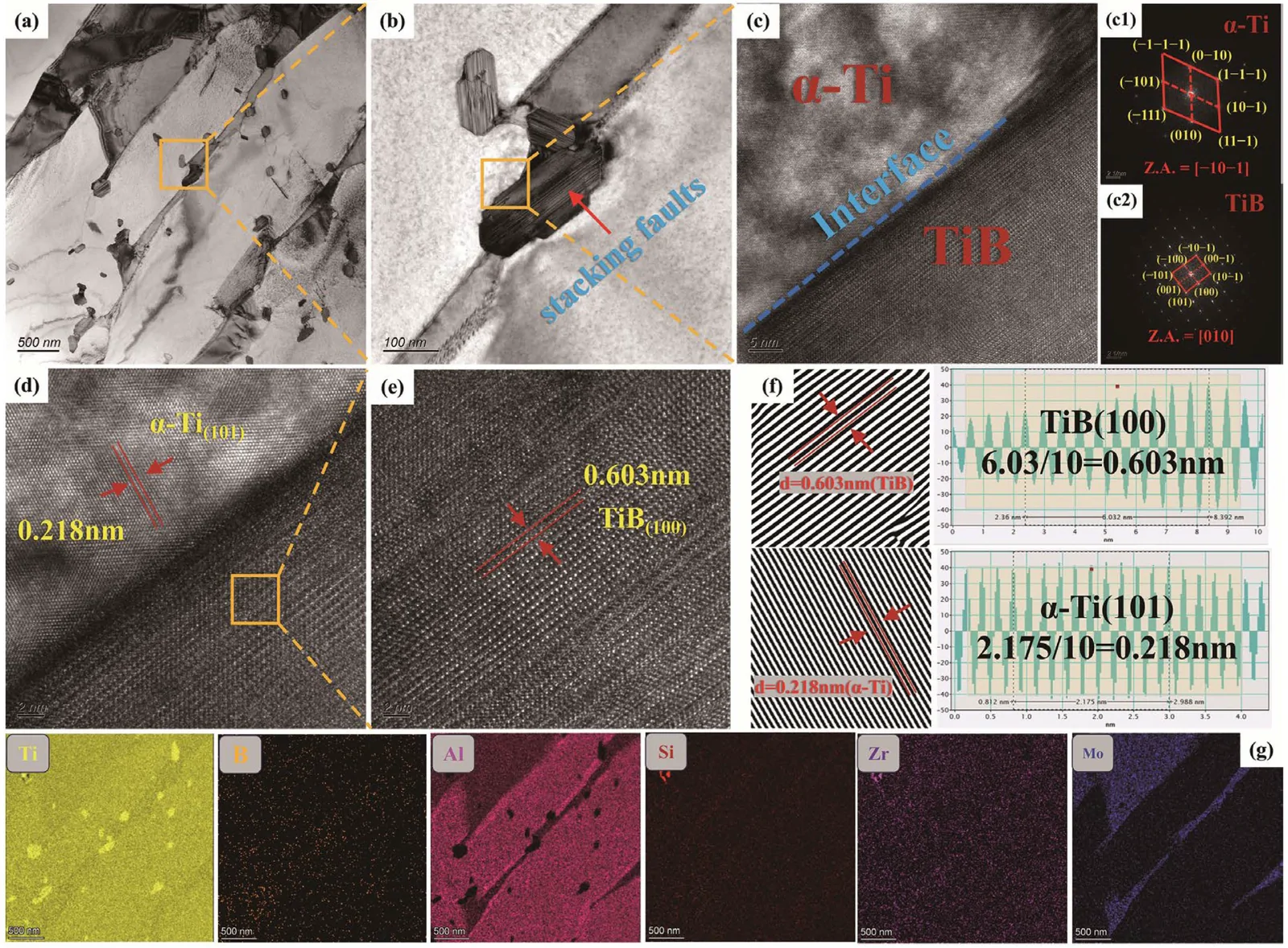
TEM characterization of polygonal in situ TiB in TMC5: (**a**) morphology of precipitated phases in TMC5, (**b**) TiB precipitates with stacking faults morphology, (**c**) HRTEM image of the TiB/α-Ti interface, (**c1**) SAED pattern of α-Ti, (**c2**) SAED pattern of TiB, (**d**) HRTEM image of α-Ti, (**e**) high-magnification HRTEM image of TiB, (**f**) inverse fast Fourier transform (IFFT) image obtained from the HRTEM image, (**g**) elemental mapping images.

Figure 7 presents the polygonal in situ TiB in TMC5. The TEM bright-field image in Figure 7a reveals that nanoscale precipitates are dispersed throughout the TC11, confirming the presence of in situ formed ceramic reinforcing phases distributed within the base alloy surrounding the network reinforcement structure. On this basis, further characterization was conducted on the nanoscale TiB precipitates, which are distinct from the elongated TiB whiskers. Polygonal and multiphase TiB precipitates can be observed in the bright-field image. Figure 7g displays the elemental mapping of Ti and B in TMC5, where the β-stabilizing element Mo is enriched in the β-Ti phase and the α-stabilizing element Al is enriched in the α-Ti phase, which is consistent with the results shown in Figure 7c. Figure 7c presents the high-resolution transmission electron microscopy (HRTEM) image of the α-Ti/TiB interface, which appears clean and well-defined, indicating a favorable interfacial bonding between the two phases. Figure 7c1,c2 show the SAED patterns of α-Ti and TiB on either side of the interface, respectively, confirming that the polygonal precipitates are the TiB phase and that the α-Ti/TiB interface is clean without any impurity phases. 

In addition, it is worth noting that a large number of stacking faults growing along the [010] direction was observed in the polygonal TiB precipitates. Due to the mismatch in the coefficient of thermal expansion (CTE) between TiB and the α-Ti matrix, thermal mismatch stress is generated during cooling, which causes local lattice distortion in TiB and subsequently induces the formation of stacking faults. Stacking faults can serve as effective barriers to dislocation motion, hindering dislocation slip and thereby increasing the yield strength of the material. Meanwhile, the faulted regions can form semi-coherent interfaces with the base alloy, enhancing the load transfer efficiency between TiB and the base alloy, and consequently improving the overall strength of the composite. Figure 7d–f present the high-resolution lattice fringe images of TiB and α-Ti, with interplanar spacings of 0.603 nm and 0.218 nm, corresponding to the (100) plane of TiB and the (101) plane of α-Ti, respectively.

**Figure 8:**
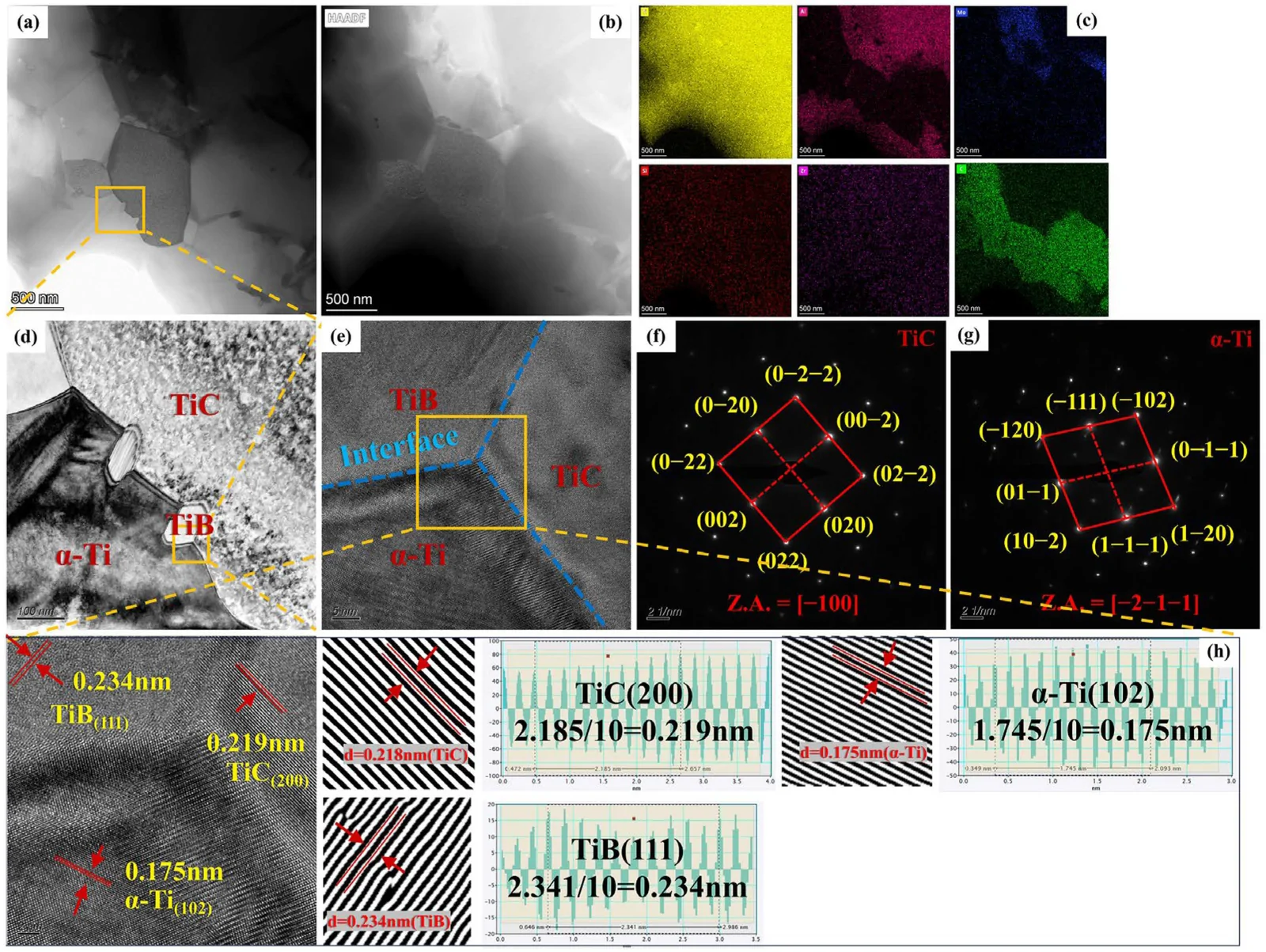
TEM characterization of symbiotic TiB and TiC in TMC5: (**a**) bright-field image, (**b**) HAADF image of the composite, (**c**) elemental mapping images, (**d**) high-magnification microstructural morphology of the selected region, (**e**) HRTEM image of the selected region, (**f**) SAED pattern of TiC, (**g**) SAED pattern of α-Ti, (**h**) HRTEM image of the selected region and its corresponding IFFT image.

Figure 8 presents the TEM characterization of symbiotic TiB and TiC in TMC5. In the bright-field image (Figure 8a), TiB exhibits dark contrast, while the α-Ti matrix appears in bright contrast. Figure 8b,f shows the high-angle annular dark-field (HAADF) image and the corresponding elemental mapping acquired from the HAADF image of the composite, respectively. It can be observed that C is enriched in the regions where the β-stabilizing element Mo and the α-stabilizing element Al are depleted, indicating that C has dissolved into the Ti, with a portion existing in solid solution to provide solid-solution strengthening and the remainder undergoing an in situ reaction with the Ti matrix to form TiC reinforcing phase. In the elemental mapping of Ti, dot-like Ti-enriched bright regions can be observed, which are highly coincident with the C-enriched regions and the Mo- and Al-depleted regions, indicating the precipitation of Ti-containing ceramic phase particles within the Ti matrix. Figure 8d presents the high-magnification TEM micrograph of the region marked by the rectangle in Figure 8a, revealing a region where TiC, TiB, and the Ti matrix coexist, with well-defined interfacial bonding observed among the phases.

Figure 8e presents the HRTEM image of the selected region, revealing a triple junction formed by the clean and well-defined interfaces among TiC, TiB, and the Ti base alloy. The SAED patterns in Figure 8f,g confirm that the Ti is α-Ti and the precipitated phase is TiC, indicating the simultaneous growth of TiC, TiB, and the Ti. Figure 8h shows the HRTEM image of the region marked by the rectangle in Figure 8e, along with the corresponding IFFT images of TiC (200), α-Ti (102), and TiB (111). Well-defined lattice fringes can be observed with interplanar spacings of 0.219 nm, 0.175 nm, and 0.234 nm, respectively. It is noteworthy that a symbiotic phenomenon exists between TiB and TiC. Previous studies have demonstrated that the intergrowth structure of TiC and TiB exhibits superior stress-bearing capability, suggesting that such a microstructure not only enhances the load-bearing capacity of the composite but also effectively hinders dislocation slip during compressive deformation [31].

The interplanar spacings measured from the HRTEM lattice fringes in Figure 6, Figure 7 and Figure 8 are consistent with the 2θ positions of the corresponding reflections in the XRD patterns (Figure 3). The phase identification by XRD and by SAED/HRTEM is therefore identical, with no additional phases detected by either technique.

## 4. Discussion

For the room-temperature property, although the reinforcing phases can enhance the yield strength through grain refinement strengthening and load transfer mechanisms, TiB whiskers and TiC particles tend to segregate along the prior β-phase grain boundaries, leading to grain boundary embrittlement and a reduction in grain boundary cohesion [11,32]. Consequently, in the high-content samples, grain boundary pores and TiB-enriched networks serve as preferential sites for crack initiation, which weakens the strengthening effect of the reinforcing phases.

For the high-temperature property, the strengthening trend of the TC11-(TiB + TiC) composites undergoes a fundamental reversal. At high temperature, severe softening occurs in the TC11 base alloy, with the peak stress dropping sharply from 1998 MPa at room temperature to 484 MPa (a reduction of approximately 75.8%), indicating that the base alloy itself has largely lost its load-bearing capacity. In contrast, the peak stress of TMC2 and TMC5 increases by 24.6% and 73.8%, respectively, relative to the base alloy, showing a positive correlation between the B_4_C content and the high-temperature strength. Owing to their excellent high-temperature modulus stability, TiB whiskers and TiC particles undergo a critical role transition from “stress concentration sites” at room temperature to a “rigid load-bearing skeleton” [11] at high temperature. As the B_4_C content increases from 2 wt.% to 5 wt.%, the volume fraction of the in situ-formed TiB whiskers increases significantly, leading to a more continuous reinforcement network in the base alloy. This network efficiently transfers external loads from the severely softened TC11 to the reinforcing phases via the shear lag mechanism [10]. Consequently, the denser and more continuous TiB network in TMC5 provides effective load-transfer paths, serving as the key microstructural mechanism for its enhanced high-temperature strength. 

Beyond the dominant load transfer described above, a secondary contribution to high-temperature work hardening may arise from the moderate coefficient of thermal expansion (CTE) differences between the reinforcements and the TC11 matrix. The CTE of the TC11 matrix is approximately 9.0 × 10^−6^ K^−1^, whereas that of TiB is about 7.2 × 10^−6^ K^−1^ [33], and that of TiC is slightly lower, in the range of 6.5–7.7 × 10^−6^ K^−1^ [34]. It shows a moderate mismatch for the TiC/matrix couple and a small one for the TiB/matrix couple. During cooling from the HIP temperature (ΔT ≈ 1125 K), these mismatches are expected to generate residual thermal stresses and interfacial defects. A simple estimate for a fully constrained matrix, σ_th_ ≈ E·Δα·ΔT (E, matrix elastic modulus; Δα, CTE mismatch between the reinforcement and the matrix; ΔT ≈ 1125 K, the HIP cooling range), gives on the order of 100–200 MPa for the TiC/matrix couple [35,36]. This is an upper bound only, since relaxation by interfacial sliding and matrix plasticity during cooling will reduce the actual stress. Direct residual-stress measurement was not available in this study, so the lattice-level deformation was assessed indirectly. The apparent XRD microstrain increases monotonically from 1.9 × 10^−3^ (TMC0) to 2.5 × 10^−3^ (TMC2) and 3.7 × 10^−3^ (TMC5), and the α-Ti (002) reflection shifts to lower angles (Section 3.1), indicating increasing lattice distortion with reinforcement content. HRTEM provides complementary local evidence. Stacking faults are observed within TiB adjacent to the α-Ti interface (Figure 7b, Section 3.3), which are attributed to thermal mismatch stress during cooling. This observation supports the expectation that the CTE mismatch induces lattice-level defects, although it does not directly quantify the residual stress field. Interfacial dislocation configurations in in situ TiB/TiC–Ti systems have also been observed by TEM in previous work [34]. During high-temperature compression, such pre-existing defects can interact with thermally activated mobile dislocations and may contribute to the higher work-hardening rate of TMC5. It should be noted, however, that the interfacial stresses generated during cooling are not static at elevated temperature. They are progressively relaxed by matrix plasticity, as discussed below.

Regarding the nature of the residual stress, the standard thermal-mismatch analysis predicts that, for reinforcements with a lower CTE, the matrix is left in tension near the interfaces while the reinforcements carry a balancing compression [35,36]. The tensile residual stress in the matrix is a double-edged factor. At room temperature, it superimposes on the stress concentration at the pores and reinforcement clusters, and it can promote crack initiation. This is consistent with the compression results: TMC5 shows the highest yield strength (1586 MPa) but the lowest fracture strain (approximately 11%) at room temperature (Figure 5a, Table 1). At high temperature, the matrix relaxes this stress by plastic flow (Section 3.3). The residual stress field therefore loses its damage potential as temperature rises, while the load-transfer skeleton remains effective, as reflected in the 73.8% increase in the peak stress of TMC5 at 650 °C.

At high temperature, the grain boundary TiB network, which acts as a crack source at room temperature, transforms into an effective load transfer channel. Meanwhile, more matrix slip systems are activated, enabling the matrix to effectively relax the stress concentration around the reinforcing phases through plastic deformation [23,37,38]. Consequently, the negative effects of grain boundary pores and TiB segregation in the high-content samples are significantly mitigated, allowing the positive strengthening effect of the reinforcing phases to be fully realized.

In addition, the B27-type orthorhombic structure of the in situ formed TiB contributes to the high-temperature performance of the composites, owing to its excellent thermal stability. The preferential growth of TiB along [010] produces the whisker morphology, while the polygonal TiB precipitates contain numerous stacking faults along [010] (Figure 7b), which act as effective barriers to dislocation motion. Furthermore, TEM and HRTEM observations confirm the intergrowth of TiB and TiC with the Ti matrix through well-bonded interfaces. Such intergrowth structures efficiently transfer loads and hinder dislocation slip during compression [31].

To make the quantitative relationships explicit, Table 1 summarizes the B_4_C content, the calculated reinforcement volume fractions, the matrix structure size, the porosity, the apparent XRD microstrain, and the compressive properties of the three composites.

## 5. Conclusions

In this work, the microstructure and mechanical properties of TC11-(TiB + TiC) composites with different B_4_C contents, fabricated by hot isostatic pressing, were systematically investigated. The main conclusions can be drawn as follows:

(1) In situ TiB whiskers and TiC particles formed in all composites, and the microstructure evolved non-monotonically with the B_4_C content. TMC2 exhibited the finest basketweave structure, whereas TMC5 showed a quasi-continuous reinforcement network, coarsened prior β grains and grain-boundary pores. The pores originate from B_4_C agglomeration and incomplete local reaction.
(2) At room temperature, the compressive yield strength increases with the B_4_C content (1020, 1327 and 1586 MPa for TMC0, TMC2 and TMC5). The peak stress and the fracture strain, however, decrease continuously. The peak stress of TMC5 (1858 MPa) is lower than that of the base alloy (1998 MPa), and the fracture strain decreases from approximately 37% (TMC0) to 11% (TMC5).
(3) At a high temperature of 650 °C, the peak stress of TC11 fell from 1998 MPa to 484 MPa. In contrast, those of TMC2 and TMC5 reached 603 MPa and 841 MPa, exceeding the base alloy by 24.6% and 73.8%.
(4) The TiB/TiC reinforcements act as stress concentration sites at room temperature but transform into a rigid load-bearing skeleton at high temperature. Through load transfer and the relaxation of interfacial stresses by matrix plasticity, the high-temperature strength increases monotonically with the reinforcement content. The moderate CTE mismatch may provide a secondary contribution, supported by the monotonic increase in the apparent XRD microstrain and by the stacking faults within TiB observed by HRTEM.
(5) The in situ TiB has a B27 structure and grows preferentially along [010], forming whisker-like morphologies. Stacking faults within TiB and the well-bonded symbiotic TiB/TiC structures hinder dislocation motion and enhance load transfer.

## Figures and Tables

**Table 1:** Quantitative summary of composition, microstructure and mechanical properties.

Parameter	TMC0	TMC2	TMC5	Data Status
B_4_C addition/wt.%	0	2	5	Nominal
TiB volume fraction/vol.%	0	≈8.8	≈21.9	In situ reaction
TiC volume fraction/vol.%	0	≈2.1	≈5.1	In situ reaction
α structure size	colonies ≈70 μm	lath width 2.5 μm	prior-β 70–130 μm	Section 3.2
Porosity/%	≈0.19	<0.3	1.1–2.7	Section 3.2
Apparent microstrain/×10^−3^	1.9	2.5	3.7	Section 3.1
RT yield strength/MPa	1020	1327	1586	Figure 5
RT peak stress/MPa	1998	1923	1858	Figure 5
RT fracture strain/%	≈37	≈22	≈11	Figure 5
650 °C peak stress/MPa	484	603	841	Figure 5

## Data Availability

The original contributions presented in this study are included in the article. Further inquiries can be directed to the corresponding author.
